# What Does the Molecular Genetics of Different Types of *Restorer-of-Fertility* Genes Imply?

**DOI:** 10.3390/plants9030361

**Published:** 2020-03-13

**Authors:** Tomohiko Kubo, Takumi Arakawa, Yujiro Honma, Kazuyoshi Kitazaki

**Affiliations:** 1 Research Faculty of Agriculture, Hokkaido University, Sapporo, Hokkaido 060-8589, Japan; 2 Gifu Prefectural Research Institute for Agricultural Technology in Hilly and Mountainous Areas, Nakatsugawa, Gifu 508-0203, Japan; 3 Department of Biotechnology and Environmental Chemistry, Kitami Institute of Technology, Kitami, Hokkaido 090-8507, Japan

**Keywords:** cytoplasmic male sterility, hybrid breeding, nuclear-mitochondrial interaction, *restorer-of-fertility*

## Abstract

Cytoplasmic male sterility (CMS) is a widely used trait for hybrid seed production. Although male sterility is caused by S cytoplasm (male-sterility inducing mitochondria), the action of S cytoplasm is suppressed by *restorer-of-fertility* (*Rf*), a nuclear gene. Hence, the genetics of *Rf* has attained particular interest among plant breeders. The genetic model posits *Rf* diversity in which an *Rf* specifically suppresses the cognate S cytoplasm. Molecular analysis of *Rf* loci in plants has identified various genes; however, pentatricopeptide repeat (PPR) protein (a specific type of RNA-binding protein) is so prominent as the *Rf*-gene product that *Rf*s have been categorized into two classes, PPR and non-PPR. In contrast, several shared features between PPR- and some non-PPR *Rf*s are apparent, suggesting the possibility of another grouping. Our present focus is to group *Rf*s by molecular genetic classes other than the presence of PPRs. We propose three categories that define partially overlapping groups of *Rf*s: association with post-transcriptional regulation of mitochondrial gene expression, resistance gene-like copy number variation at the locus, and lack of a direct link to *S-orf* (a mitochondrial ORF associated with CMS). These groups appear to reflect their own evolutionary background and their mechanism of conferring S cytoplasm specificity.

## 1. Introduction

Cytoplasmic male sterility (CMS) is a genetic character that prevents plants from producing functional pollen [1]. In most cases, CMS affects nothing but the male reproductive organ; however, some pleiotropic effects are known such as disease susceptibility and flower morphology (e.g., narrow petals) [2,3]. Due to its maternal inheritance, CMS is widely used in plant breeding to prepare seed parents for hybrid-seed production [1]. In other cases, CMS is a potential obstacle for crossing between a breeding line and genetic resources [4,5,6]. Controlling CMS expression is one of the major challenges in plant breeding.

In the widely held genetic model, the male-sterility inducing cytoplasm is referred to as S cytoplasm, whereas cytoplasm without the male-sterility inducing factor is termed N cytoplasm. As was shown in some initial genetic analyses (e.g., [7]), CMS is governed not only by S cytoplasm but also is affected by nuclear genes because offspring from a CMS plant do not always express male sterility depending on the pollen parental line. Differences among such nuclear genotypes cannot be seen in the presence of N cytoplasm, hence a genetic interaction between S cytoplasm and a nuclear gene is postulated. In many cases, a nuclear suppressor gene is proposed as a *restorer-of-fertility* in the genetic model where a dominant allele (*Rf*) suppresses S. An *Rf* affects nothing when expressed in a different source of S cytoplasm, indicating that the *Rf* specifically suppresses the cognate S. This observation means that differences in S cytoplasm can be defined by differences in *Rf* [8]. Note that *Rf* genes have been named in each plant species; consequently, maize *Rf2* (*Rf2a*) and rice *Rf2* are not orthologous genes. Unraveling the genetics of *Rf* is a prerequisite for the efficient use of CMS in hybrid seed production, otherwise discerning the *Rf* genotype for plant breeding would be laborious [9].

Molecular analysis of CMS has associated the S cytoplasm with mitochondrial open reading frames (ORFs) composed of pieces of duplicated genes and/or origin-unknown sequences [10,11,12], whereas other studies show associations with truncated mitochondrial genes, mitochondrial non-coding RNAs, and over-expression of mitochondrial genes [13,14,15]. Plants that have lost S cytoplasm-associated ORFs (hereafter *S-orf*s) from the mitochondrial genome have been obtained by mutation, somaclonal segregation or mitochondrial genome editing [10,16,17]. The phenotypes of these plants indicate that *S-orf* is responsible for male sterility but plays no other role in plant development.

The identified *S-orf*s differ in their primary sequences [11], hence knowing the nucleotide sequence of a mitochondrial genome is insufficient for identifying an *S-orf*. Nonetheless, alteration of *S-orf* expression in a fertility-restored plant by *Rf* is frequently observed [10]. Based on this finding, a common approach for identifying *S-orf*s is to find an alteration in gene expression of a mitochondrial gene/ORF by *Rf*. Note that, although this approach is broadly applicable for CMS study, there are some exceptions as discussed below.

Since the first molecular cloning of *Rf* [18], a number of *Rf* loci have been analyzed to identify *Rf*s or *Rf* candidates. As reports increase, *Rf*-gene products are now known to be numerous and variable (Table 1). We raise the question as to whether each of these *Rf*s is an idiosyncratic example that episodically emerged during plant-species diversification or is there a tendency or trend underlying *Rf* evolution. To approach this question, we will briefly review plant *Rf*s. Recent reviews on CMS, *Rf* and other types of male sterility will help to understand the progress of this research area [19,20,21,22,23,24,25,26,27,28,29,30,31,32]. This question has also been addressed from the viewpoint of evolutionary genetics (reviewed ib [33]). 

## 2. Pentatricopeptide Repeat Protein is the Most Abundant Type of *Rf* Gene Product

Pentatricopeptide repeat (PPR) proteins have a succession of degenerate ~35 amino acid sequence motifs with the potential for binding to single-stranded RNA in a nucleotide-sequence specific manner [45]. PPR protein genes are known to form one of the largest gene families among land plant genomes [45]. In most cases, PPR protein gene products are thought to be imported into mitochondria, plastids, or both [45]. PPR protein genes play pivotal roles mainly in post-transcriptional mechanisms and are subdivided into several classes based on the length of the PPR motifs and the presence/absence of additional domains [45]. According to this classification, most of the *Rf*s encoding PPR proteins (hereafter PPR *Rf*) belong to the P class (reviewed in [34,35]), in which canonical repeats occupy almost the entire coding region [45]. To date, the P-class PPR protein is the most frequently reported class of *Rf*-gene product.

The molecular action of most PPR *Rf*s involves a reduction in S-ORF-protein accumulation in the cognate S cytoplasm [34]; however, details of the action differ among plants. For example, altering the transcript profile of the *S-orf* is the case for rice *Rf1a* but not for radish *Rfo/Rfk* [34]. Additionally, in spite of the notion that PPR protein has the potential to bind RNA, gene products of PPR *Rf* do not necessarily bind with the cognate *S-orf* RNA even when accumulation of the S-ORF protein is reduced [34]. Another factor has been reported to mediate between PPR RF protein and *S-orf* mRNA [46].

Sorghum *Rf1* and barley *Rfm1* are associated with a PLS-DYW-class PPR protein, where long- and short versions of PPR motifs are included in the repeat array and an extra domain, termed DYW, is added at the carboxyl terminus [36,37]. As PLS-DYW-class PPR proteins almost exclusively play a role in RNA editing, a post transcriptional process that converts specific cytidine residues into uridine [47], it is possible that sorghum *Rf1* and barley *Rfm1* restore pollen fertility by editing *S-orf* transcripts or other target RNAs that are currently unknown. Molecular analysis of the cognate S cytoplasm is necessary.

## 3. What Do *Rf*s Encode Other than PPR Proteins?

The interaction between nuclei and mitochondria is very complex in plants, as it is in other eukaryotes [48]. Hence, it may be not surprising that some *Rf*s encode proteins other than PPR proteins. We refer to such *Rf*s as non-PPR *Rf*s. 

Maize *Rf2a* is the first *Rf* whose gene product was identified and shown to encode a mitochondrial aldehyde dehydrogenase (ALDH) [18,42]. Its recessive alleles (i.e., male-sterility inducing alleles) have a transposon insertion or a missense mutation that reduces the level of gene expression or abolishes enzymatic activity, respectively [42]. Maize *Rf2a*, in conjunction with *Rf1*, restores male fertility to plants with CMS-T, one of the three S cytoplasms in maize [10]. The *S-orf* of CMS-T is identified as a chimeric ORF consisting of different parts of *rrn26*, but *Rf2a* does not affect transcripts or protein products of this *S-orf* [10]. The target molecule of *Rf2a* in anthers is unknown, but recombinant RF2A protein expressed in *E. coli* can oxidize a broad range of aldehydes [49].

Maize *Rf4* is a restorer of another maize S cytoplasm called CMS-C [43]. Its gene product is a basic helix-loop-helix (bHLH) transcription factor [43]. Surprisingly, the RF4 protein has a nuclear-localization signal, suggesting that the protein may localize outside of mitochondria [43]. This finding appears to discount the prevailing notion that *Rf* gene products are all imported into mitochondria. The identity of the *S-orf* in CMS-C mitochondrial genomes is unknown, although three candidates have been found (discussed by Allen et al. [50]), making the mechanism for how *Rf4* restores pollen fertility more elusive. Maize *Rf4* is an allele of *Ms23* that was originally identified as playing an essential role in the differentiation of tapetal tissue in anthers [51]. Null alleles of *ms23* cause male sterility even in N cytoplasm plants, whereas the *rf4* allele (inducing male sterility in CMS-C but male fertile in N cytoplasm) has an amino acid substitution that is potentially harmful to the stability of a heterodimer composed of RF4 and another protein bHLH51 [43]. The male-sterility inducing *rf4* allele appears to be leaky.

Rice *Rf17* encodes a protein that is partially homologous to an acyl-carrier protein synthase that is likely imported into mitochondria [44]. Although a candidate *S-orf* was identified, no alteration in the transcript profile was observed [52]. *Rf17* restores pollen fertility in a gametophytic manner (i.e., those pollen grains receiving a restoring allele are fertile but otherwise sterile). Molecular analysis associated a reduction in the expression of the restoring allele with fertility restoration [44]. This notion was confirmed by an observation that ectopic expression of RF17 protein canceled fertility restoration [44]. A working hypothesis is that RF17 transmits a retrograde signal that is emitted from S mitochondria to exert male sterility, but the reduced expression of *Rf17* results in the blockage of this transmission [44]. The allelic difference is attributed to a single nucleotide substitution in the region upstream of the start codon [44].

In contrast to the abovementioned *Rf*s, some non-PPR *Rf*s are known to or potentially affect *S-orf* expression in different ways. Rice *Rf2* reduces the amount of dicistronic mitochondrial transcripts encoding *atp6* and *orf79* (an *S-orf* of the cognate S cytoplasm), whereas monocistronic *atp6* mRNA remains unaffected, resulting in the reduction of ORF79 protein accumulation [53]. Positional cloning of rice *Rf2* revealed that this gene encodes a protein having a characteristic glycine-rich region [38], but whether there is molecular interaction between RF2 protein and *orf79* transcript is unclear [53]. The male-sterility inducing allele is expressed to a level comparable to that of the restoring allele, but it has a single amino acid substitution [38].

Barley *Rfm3* is genetically associated with two linked genes encoding mitochondrial transcription termination factor family (mTERF) proteins [39]. Interestingly, rye *Rfp1* shows tight linkage with an mTERF protein gene [40]. mTERF protein, as well as PPR protein, belongs to a large family of helical-repeat proteins capable of binding to nucleic acids [54]. The copy number of mTERF protein genes in plants exceeds that of vertebrates; at least 33 and 28 mTERF protein genes have been identified in the *Arabidopsis* and rice genomes, respectively, whereas only four mTERF protein genes are found in human and mouse [55]. A few of the plant mTERF protein genes have been characterized; their gene products are imported into mitochondria or plastids to bind DNA or RNA and are involved in transcription, splicing, or tRNA maturation [54]. Further study is necessary to elucidate the identity and targets of *Rfm3* and *Rfp1*.

Sugar beet *Rf1* is a duplicated gene of *Oma1* [41,56]. *Oma1* (named after **o**verlapping activity with **m**-**A**AA protease) is known to be involved in quality control of mitochondria and mitochondrial dynamics in yeast and mammals [57,58,59]. Sugar beet S cytoplasm is associated with an *S-orf* termed *preSatp6* whose origin is unknown [60]. There is no observable effect of *Rf1* on the transcript size or the amount of *preSatp6* translation product [60,61]; however, whereas preSATP6 protein is detected from a 250-kDa complex (presumably a homo-oligomer form) in CMS anthers, the 250-kDa complex disappears in fertility restored anthers and, instead, novel complexes appear [60]. RF1 protein expressed from a restoring allele can bind to preSATP6 protein, but the gene product from a non-restoring allele does not show such activity [60]. The *Rf1* appears to alter the higher order structure of preSATP6 protein without degradation, as do some molecular chaperones. These findings also imply that a higher order structure of preSATP6 protein is an important factor for CMS expression, whereas the preSATP6 protein *per se* may be less harmful for pollen production [60].

## 4. How Have *S-orf* and *Rf* Evolved?

An organizational comparison between S- and N mitochondrial genomes and between *Rf* and *rf* alleles has been conducted to identify associated genes and to identify the molecular basis of relevant DNA markers. The results have invoked studies on the evolutionary aspects of *S-orf* and *Rf*.

### 4.1. Evolution of S-orf

The size of plant mitochondrial genomes ranges from 66 kbp to 11.3 Mbp but is usually 200–700 kbp or more than 12 times larger than mammalian counterparts [25,62]. Despite their large size, the number of plant mitochondrial genes is 50–60, less than twice that of mammals [25]. Hence, the plant mitochondrial genome is referred to as a gene sparse genome, a term that means the existence of large intergenic regions [25]. Most *S-orf*s occur in the vicinity of a mitochondrial gene (in many cases, genes encoding subunits of ATP synthase) [11]. *S-orf*s often exhibit a chimeric nature in which pieces of mitochondrial gene fragments are joined together [10]. The chimeric nature of *S-orf* reflects the principal mechanism for plant mitochondrial genome diversification in which frequent inter- and intra-molecular recombination plays a very important role [25]. In fact, duplication of DNA segments and rearrangement of the mitochondrial genome between closely related species or even within species can be explained by this mechanism [25]. It is likely that *S-orf*s independently evolved in each plant lineage as by-products of plant mitochondrial evolution.

### 4.2. Evolution of PPR Rfs

The PPR *Rf* locus frequently contains arrayed gene copies that are highly similar to PPR *Rf* [35], suggesting the occurrence of local gene duplication. Genes having similarity with PPR *Rf* are designated *restorer-of-fertility like* (*RFL*) genes [63]. Organizational differences between restoring and non-restoring alleles of a PPR *Rf* locus often involve copy number variation (CNV) of *RFL* genes; detailed comparison between the alleles revealed traces of interallelic recombination with unequal crossing over [34]. Such an evolutionary mechanism could result in the production of multiple, different molecular variants with various numbers of *RFL* copies within an *Rf* locus. In fact, analysis of the rice *Rf1* (a typical PPR *Rf*) locus using 59 accessions of *Oryza* genetic resources revealed the existence of at least six molecular variants with CNV [64].

*RFL* and PPR *Rf* (except for those encoding PLS-DYW class proteins) constitute a unique subgroup within the P-class of PPR genes. *RFL* is almost ubiquitous in plant genomes even when the plant appears to have no relationship to CMS, such as poplar (a dioecious plant species) [63,65,66,67]. *RFL* copy number in a genome varies among plant species (sometimes close to 40 copies) [68]. *RFL* copies tend to cluster with each other in several chromosomal regions, but the location of the cluster is less preserved even between closely related species. For this reason, *RFL* is said to be “nomadic” in evolutionary scale [69]. According to a phylogenetic analysis, *RFL* already existed before the split between monocot- and dicot lineages. The current *RFL* members form species-specific clusters that are paralogous to each other, a similar evolutionary pattern observed in pathogen resistance (*R*) genes [34].

*RFL* is thought to participate in the quality control of the transcript pool in plant mitochondria [34]. In plant mitochondrial genomes, many ORFs that are obviously distantly related or unrelated to genuine mitochondrial genes occur in the large intergenic regions [70]. As transcriptional control in plant mitochondria is relaxed [34], potentially harmful RNAs including those encoding aberrant proteins can be generated [71]. A post-transcriptional control system to cope with such harmful RNA is employed by plant mitochondria, and *RFL* is an important component of this system. The emergence of PPR *Rf* from *RFL* is a very likely scenario.

### 4.3. Evolution of Non-PPR Rfs

The organizational diversity of non-PPR *Rf*s in genetic resources is less investigated. Several haplotypes based on single nucleotide polymorphisms and small insertion/deletions were identified in two linked mTERF protein genes at the barley *Rfm3* locus, and one haplotype is exclusively found from a non-restoring genotype [39]. 

CNV within a non-PPR *Rf* locus has rarely been reported to date. An exceptional case is the sugar beet *Rf1* locus, in which *Rf1*-like genes are tandemly clustered [56], reminiscent of the PPR *Rf* locus. In fact, CNV is seen in the *Rf1* locus of sugar beet and other *Beta vulgaris* genetic resources (e.g., Arakawa et al. [72]), suggesting that a similar evolutionary mechanism to that of PPR *Rf* may be involved (i.e., interallelic recombination with unequal crossing over).

The genetic function of multiple *Rf1* molecular variants is largely unknown. A handful of these variants have been subjected to genetic analysis, and semi-dominant- (fertility restoration is insufficient when heterozygous) and hypomorphic (the protein product has the potential to bind with preSATP6 protein but barely restores fertility due perhaps to an insufficient amount of mRNA) alleles were identified, in addition to dominant and recessive alleles [72,73]. This observation indicates multiple allelism of the *Rf1* locus. The number of beet *Rf1* molecular variants is increasing as more *B. vulgaris* genetic resources are investigated [74]. One could raise the question of the significance of a series of different strength *Rf*s.

The clustered genes in the sugar beet *Rf1* locus were designated *RF1-Oma1* [56]. From the sugar beet genome, another *Oma1* homologue that is more similar to *Arabidopsis Oma1* (*atOma1*) was found and named *bvOma1*. In yeast, a Zn^2+^-binding motif in the peptidase M48 domain was shown to be critical for *Oma1* function [57]. The peptidase M48 domain with a functional Zn^2+^- binding motif was predicted from the translation products of *atOma1* and *bvOma1* but not from *RF1-Oma1* due to a critical amino acid substitution [56]. The occurrence of multiple *Oma1* homologues in the sugar beet genome contrasts with *Arabidopsis* and rice, both of which have a single *Oma1* copy as is also the case for yeast and humans [41]. An *Arabidopsis* plant with a defect in *atOma1* is viable but has malfunctioning oxidative phosphorylation [75]. A comparison of the proteins’ ability to bind with preSATP6 and the expression pattern among *RF1-Oma1*, *bvOma1* and *atOma1* is summarized in Table 2; neither *bvOma1* nor *atOma1* had binding activity with preSATP6 protein [56]. The expression pattern during anther development was different among the three genes, of which only *RF1-Oma1* was expressed at the meiosis stage [56]. Our unpublished data indicated that *RF1-Oma1* is likely a paralogue of *Oma1* [76]. Taken together, these results suggest that neofunctionalization may be favored to explain beet *Rf1* evolution in which an ancestral *Oma1* (possibly represented by *atOma1*) was duplicated, then the duplicated copy acquired several novel functions to evolve into *RF1-Oma1*.

Neofunctionalization might be, however, an over-simplification considering the difference in expression patterns between *bvOma1* and *atOma1*; the difference is characterized by the lack of expression at the tetrad stage in *bvOma1* (Table 2). Interestingly, *RF1-Oma1* is expressed at the tetrad stage, which appears to complement this missing expression. Another molecular evolutionary mechanism posits that two duplicated genes can escape from evolutionary decay when each has a unique expression pattern and they complement each other to fulfil the expression pattern of their parental gene [77]. Whether the apparent degenerative expression pattern in *bvOma1* is involved in the evolution of *RF1-Oma1* is unknown. The important question is whether *RF1-Oma1* has functions other than altering the higher order structure of preSATP6 protein. Concerning this question, the organization of *rf1* alleles from different origins was investigated by analyzing sugar beet lines selected for the non-restoring genotype [78]. Interestingly, all genotypes had preserved, intact *RF1-Oma1* copies despite lacking the ability to bind with preSATP6 protein [60,78], strongly suggesting an unknown function of *RF1-Oma1*. A more detailed study will be necessary.

## 5. Conclusions and Perspectives

The molecular genetics of *Rf* has been deepened by the studies on PPR *Rf*. Conversely, studies on non-PPR *Rf* have shed light on aspects that cannot be investigated by examining PPR *Rf*. Clearly, both types of studies are complementary to each other and are essential for understanding *Rf* and S cytoplasm function.

Non-PPR *Rf* now becomes a group of miscellaneous gene products; however, it seems possible to find some shared features between different types of *Rf*s or with PPR *Rf*. Our present investigation is to introduce new criteria into *Rf* grouping instead of that solely based on PPR. As shown below, we propose three groups that are not necessarily mutually exclusive (Table 1). Some remaining questions are also posed.

**Group 1: Association with a Post-Transcriptional Mechanism for Regulating Mitochondrial Gene Expression.**

No doubt, the number of *Rf*s associated with a post-transcriptional mechanism is highest due to the large number of PPR *Rf*s [34,35]. Some non-PPR *Rf*s are also associated with post-transcriptional mechanisms such as in rice *Rf2* [38]. It is possible that barley *Rfm3* is associated with this mechanism by analogy with mTERF protein function (this might also be the case for rye *Rfp1*) [39,40,54], but identifying the mitochondrial gene responsible for CMS and studying the effect of *Rf* at the molecular level is necessary.

The prevalence of this group in *Rf* is reminiscent of genomic and proteomic studies in which a large portion of the plant mitochondrial proteome is composed of proteins capable of RNA binding or RNA processing, suggesting the importance of post-transcriptional mechanisms in plant mitochondria [47]. The abundance of genes for such proteins could be a rich resource for *Rf* evolution.

**Group 2: *R* Gene-Like CNV at the Locus.**

Loci of PPR *Rf* and sugar beet *Rf1* (encoding OMA1-like protein) exhibit CNV in genetic resources [34,35,64,72]. This pattern of diversification is analogous to the *R* gene that evolves to convey pathogen resistance, whereas the pathogen evolves to overcome the *R* gene, a situation referred to as the “arms race” [63,79]. In terms of evolutionary genetics, mitochondrial and nuclear genomes are two conflicting parties because the former is inherited maternally, meaning that the male gamete is useless for mitochondria, whereas the male gamete is an important vehicle for the bi-parentally inherited nuclear genome. Hence, whereas mitochondria welcome CMS, the nuclear genome responds by implementing *Rf* [80]. 

*R* gene products usually detect pathogen elicitors in either a direct or indirect way to initiate a defense response [81]. Note that specific physical interaction between an RF protein and an *S-orf* transcript or the protein is the key to the fertility restoration of Group 2 members, such as some PPR *Rf* and sugar beet *Rf1* [60,82,83]. Possibly, the role of Group 2 members is to detect male-sterility inducing factors, analogous to the relationship between the *R* gene and an elicitor. However, reports on rice *Rf5* (identical to rice *Rf1*) and *Rf6* (encoding a P-class PPR protein) in which the two *Rf*s showed no binding activity with the cognate *S-orf* [46,84] are the caveats of this proposed function. It is currently unknown what shapes the CNV at an *Rf* locus.

**Group 3: Lack of a Direct Link with *S-orf*.**

This group includes maize *Rf2a*, maize *Rf4* and rice *Rf17*, all of which lack evidence demonstrating that they affect *S-orf* transcripts or proteins. Note that the *S-orf* is uncharacterized in maize CMS-C (the cognate S cytoplasm of *Rf4*) and rice CMS-CW (that of *Rf17*). 

Interestingly, the null alleles of maize *rf2a* generated by transposon mutagenesis greatly reduce male fertility when combined with an N cytoplasm [42]. Another recessive allele of maize *rf2a* recovered from a breeding line preserved an intact ORF that was expressed to the level of the dominant allele but had amino acid substitutions [42]. These findings parallel the relationship between *ms23* and *rf4*, the former is a null allele causing tapetum abnormality and the latter is a missense allele in the same locus [43]. These results suggest that, in this group, recessive alleles that can be used for CMS expression but should secure pollen production with an N cytoplasm might be weak alleles at the molecular level. The role of such weak alleles possibly may be increasing anther susceptibility to the S cytoplasm for CMS expression. If so, the *Rf*s of this group would be a useful tool for determining how the S cytoplasm induces male sterility. The link between *S-orf*s and the *Rf*s of this group will be more obvious following a detailed physiological and developmental study.

A puzzling question remains. In the genetic model, *Rf* specifically suppresses the cognate S cytoplasm (hence the *Rf* can be used to diagnose S cytoplasm) [8]. Given that the molecular basis of S cytoplasm is associated with *S-orf*, the differences in S cytoplasms are attributable to the differences in *S-orf*s. In the cases where specific binding of RF protein with an *S-orf* transcript or the protein is critical for fertility restoration (e.g., some *Rf*s in Group 2), the genetic specificity of an *Rf* to the cognate S cytoplasm is easily explained. However, such data are missing in many cases, and the question of why an *Rf* specifically restores the cognate S cytoplasm remains unanswered. This issue is conspicuous in the cases of Group 3 *Rf*s. Possibly, each S cytoplasm has a unique mechanism to induce male sterility, and the *Rf* arbitrarily targets one of the steps within the entire cascade. Alternatively, the mechanism for male-sterility induction is generally common among S cytoplasms [85], but one (or some) of the steps within the entire cascade may be uniquely vulnerable in each S cytoplasm and is, thus, exposed as a target of *Rf*.

In conclusion, some of the different types of *Rf*s can be grouped by molecular genetic features other than PPR. The reason why these features work is not well understood, but the involvement of their evolutionary background is possible. Cross-disciplinary studies including molecular genetics, evolution, physiology and developmental studies will be necessary to refine the details of *Rf* and CMS. The outcome will be beneficial to plant breeding where controlling CMS is highly desired.

## Figures and Tables

**Table 1:** Summary of protein products of *restorer-of-fertility* (*Rf*) and *Rf* candidate.

Gene Product ^1^		Post-transcriptional Mechanism in Mitochondria	*R* Gene-like CNV at the Locus	Direct Link with *S-orf*	References
PPR protein	P-class	Yes	Yes	Yes	[34,35]
	SPL-DYW	Undetermined	Undetermined	Undetermined	[36,37]
GR protein		Yes	Undetermined	Undetermined	[38]
mTERF protein		Undetermined	Undetermined	Undetermined	[39,40]
OMA1-like protein		No	Yes	Yes	[41]
ALDH		No	No	No	[18,42]
bHLH		No	No	No	[43]
ACP-like protein		No	No	No	[44]

^1^ PPR, pentatricopeptide repeat protein; GR, glycine-rich; mTERF, mitochondrial transcription termination factor family; OMA1, a gene symbol named after overlapping activity with m-AAA protease; ALDH, aldehyde dehydrogenase; bHLH, basic helix-loop-helix transcription factor; and ACP, acyl-carrier protein synthase.

**Table 2:** Differences in protein function and expression patterns of *Oma1* homologues in sugar beet and *Arabidopsis* ^1^.

Gene Name ^2^	Protein Product to Bind with PreSATP6	mRNA Detection			
		Tissue	Anther developmental stage		
			Meiosis	Tetrad	Microspore
*RF1-Oma1*	Yes (dominant allele)/ No (recessive allele)	Tapetum	+	+	-
		Meiocyte/Tetrads/ Microspore	+	+/-	-
*bvOma1*	No	Tapetum	-	-	+
		Meiocyte/Tetrads/ Microspore	-	-	+
*atOma1*	No	Tapetum	-	+	+
		Meiocyte/Tetrads/ Microspore	-	+	+

^1^ Based on [55]. ^2^ *RF1-Oma1*, *Oma1*-like genes clustered in the sugar beet *Rf1* locus; *bvOma1* and *atOma1*, probable *Oma1* orthologues of sugar beet and *Arabidopsis*, respectively.
